# A New Comorbidity in Periodontitis: *Fusobacterium nucleatum* and Colorectal Cancer

**DOI:** 10.3390/medicina58040546

**Published:** 2022-04-15

**Authors:** Francisco Mesa, Maria José Mesa-López, Juan Egea-Valenzuela, Cristina Benavides-Reyes, Luigi Nibali, Mark Ide, Giuseppe Mainas, Manfredi Rizzo, Antonio Magan-Fernandez

**Affiliations:** 1 Department of Periodontics, School of Dentistry, University of Granada, 18071 Granada, Spain; fmesa@ugr.es; 2 Gastroenterology Service, Virgen de la Arrixaca University Hospital, 30120 Murcia, Spain; mari_ml_2@hotmail.com (M.J.M.-L.); juanegeavalenzuela@gmail.com (J.E.-V.); 3 Department of Operative Dentistry, School of Dentistry, University of Granada, 18071 Granada, Spain; crisbr@ugr.es; 4 Periodontology Unit, Centre for Host Microbiome Interactions, Faculty of Dentistry, Oral & Craniofacial Sciences, King’s College London, London SE1 9RT, UK; luigi.nibali@kcl.ac.uk (L.N.); mark.ide@kcl.ac.uk (M.I.); giuseppe.mainas@kcl.ac.uk (G.M.); 5 Department of Health Promotion, Mother and Child Care, Internal Medicine and Medical Specialties, School of Medicine, University of Palermo, 90133 Palermo, Italy; manfredi.rizzo@unipa.it

**Keywords:** periodontitis, dysbiosis, *Fusobacterium nucleatum*, colorectal cancer

## Abstract

There is very recent and strong evidence relating *Fusobacterium nucleatum* to colorectal cancer. In this narrative review, we update the knowledge about gingival dysbiosis and the characteristics of *Fusobacterium nucleatum* as one of the main bacteria related to periodontitis. We provide data on microbiome, epidemiology, risk factors, prognosis, and treatment of colorectal cancer, one of the most frequent tumours diagnosed and whose incidence increases every year. We describe, from its recent origin, the relationship between this bacterium and this type of cancer and the knowledge and emerging mechanisms that scientific evidence reveals in an updated way. A diagram provided synthesizes the pathogenic mechanisms of this relationship in a comprehensive manner. Finally, the main questions and further research perspectives are presented.

## 1. Introduction: Dysbiosis of the Subgingival Microbiome

Human microbiomes are polymicrobial structures, and the different microenvironments that harbour them are regulated by environmental factors, complicated inter-microbial communication signals, and hosts’ immune responses. A collective functioning of these microbial communities leads to symbiosis or eubiosis with the host. However, when they are broken due to the dominance of specific species, it leads to dysbiosis and the onset of pathological processes or diseases [1]. In the oral microbiome, extrinsic factors such as diet, tobacco, stress, and antibiotics, as well as intrinsic factors such as poor dental hygiene, cytokines, microRNAs, and diabetes, will have an effect on the microbiota-host immunity axis, promoting the loss of microbial diversity of the symbiotic subgingival biofilm, and favouring the predominance of a certain disease-initiating dysbiotic flora. It has been now accepted that periodontitis is caused by an imbalance in the subgingival microbial composition and function (dysbiosis) rather than an infectious process caused by specific periodontopathogenic species [2]. Therefore, the microbiota associated with a state of health is considered more varied and stable over time; whereas the microbiota associated with disease is dominated by ‘specialist’ microorganisms that have metabolic functions and high virulence potential that are adapted to and promote the persistence of a disease state [3,4].

The subgingival microenvironment is characterized by being exposed to the gingival crevicular fluid and to low oxygen pressures when it comes to gingival pockets (patients with periodontitis). This fact favours the accessibility and/or colonization of specific microbial communities (strict anaerobes) that could cause a homeostatic imbalance in the hosts (dysbiotic biofilm) and induce a destructive inflammatory process, affecting the hosts’ immune systems (ecological plaque hypothesis). These characteristics support the evidence that, in patients with periodontitis, the gingival microbiome becomes less diverse and this diversity is also lower in the gastrointestinal tract [5], probably due to the additional nutrients provided by tissue damage (destruction of bones and connective and epithelial tissue) and the physical conditions of deeper and deeper closed spaces (periodontal pockets) [1]. This periodontal dysbiotic biofilm seems to interact with inflammation, as recently proposed by the inflammation-mediated polymicrobial-emergence and dysbiotic-exacerbation (IMPEDE) model, considering inflammation as the main agent, shifting the microbial environment and contributing to disease progression [6]. Anti-inflammatory treatments have not only inhibited periodontitis in mice, rats, and rabbits, but they have also decreased the periodontal bacterial load and reversed dysbiosis [7]. Finally, transcriptomic analyses of the biofilm associated with periodontitis have revealed high expression of genes related to proteolytic enzymes, genes related to peptide transport and iron acquisition, and genes for the synthesis of lipopolysaccharides, all of them demonstrating the clear pro-inflammatory potential of this biofilm [8].

These perturbations in the subgingival microbiome leading to periodontitis have been found in both young and elderly cohorts of periodontal patients [9], but periodontal treatment and hygiene measures have also shown to have an effect in reverting this perturbation. An example of how dental hygiene can influence the disruption of interdental biofilm through the use of interdental brushes has been recently demonstrated by Bourgeois et al. in a clinical trial. They assessed 100 interproximal spaces in 25 healthy young individuals, comparing spaces sanitized using interproximal brushes with others without cleaning. These authors demonstrated, at different times, using rtPCR, that a decrease in red complex bacteria (Socransky) (*Porphyromonas gingivalis, Treponema denticola,* and *Tannerella forsythia*) and orange complex bacteria (*Fusobacterium nucleatum, Prevotella intermedia, Prevotella nigrescens, Parvimonas micra, Eubacterium nodatum*, and *Campylobacter rectus*) is considered a high and moderate risk for periodontitis according to conventional models based on bacterial cultures. A symbiotic favourable microbiota was re-established (yellow, blue, and purple Socransky bacterial complexes), and, clinically, inflammation in interdental sites decreased after three months, when compared to the initial state [10]. This same improvement in microbiological factors was observed even in in the absence of professional periodontal treatment, in patients trained to perform a proper subgingival root brushing [11].

However, there is another scientific trend that questions: (a) the role of subgingival dysbiosis in periodontitis, i.e., the determining role of age and the hyper-responsiveness of neutrophils in late forms of periodontitis in sensitive adults; (b) the positive experimental response in oral health with anti-aging therapies [12]; (c) the change that occurs in the oral microbiota due to dysregulated immunity [13].

## 2. Fusobacterium Nucleatum

One of the most representative pathogenic species of the subgingival dysbiotic biofilm is *Fusobacterium nucleatum* (Fn). The pathogenic species of the genus *Fusobacterium* spp. include *F. nucleatum*, *F. necrophorum*, *F. canifelinum*, *F.gonidiaformans*, *F. mortiferum*, *F. naviforme*, *F. necrogenes*, *F. russii*, *F. ulcerans*, and *F. varium*, the first two species being the most pathogenic [14].

Fn is an anaerobic, invasive, and pro-inflammatory bacterium, mainly linked to periodontitis but also associated with odontogenic abscesses and other oropharyngeal diseases such as Lemierre’s syndrome [15]. It is rare in the faecal microbiome and, until recently, never described in colon adenocarcinomas. With an activity similar to *Porphyromonas gingivalis*, it causes a stimulation of IL-8 (pro-inflammatory cytokine) ten times longer than that caused by *E. coli*. It expresses numerous adhesins, such as RadD, by which it binds to the SpaP adhesin of Streptococcus mutans and Candida albicans in coaggregation and organization phenomena of the oral biofilm [16,17]. However, three characteristics make Fn a particularly pathogenic bacterium. Fn adhesin A (FadA), expressed on the surface, makes epithelial and endothelial cells adhere and invade, binding to E-cadherin (transmembrane adhesion glycoprotein, responsible for cell-cell union) [18]. Vascular endothelial-cadherin, a protein responsible for cell-cell junction at the endothelial level, was determined as the endothelial receptor for FadA. FadA co-localized with cadherin on endothelial cells caused the relocation of cadherin away from the cell-cell junctions [19]. As a result, endothelial permeability was increased by loss of the intercellular union, allowing the bacteria to cross the endothelium. This crossing mechanism may explain why the organism is able to disseminate systemically to colonize different body sites [20]. FadA is unique to Fn and exists in two forms, namely, non-secreted pre-FadA, bound to the inner surface of the bacterial membrane, and secreted mature mFadA (125 aa protein) that is expressed on the bacterial surface. Together, mFadA and pre-FadA form a high molecular weight complex, necessary for attaching and invading host cells [21]. Lectin-type Fap-2 molecules exhibit a high affinity to bind to polysaccharides that express tumours (d-galactose-β (1–3)-N-acetyl-d-galactosamine (Gal-GalNAc) [22] and exhibit the ability to block the action of immune cells. Finally, the universal autoinducer molecule (AI-2) mediates the intergenetic signalling of multiple species in bacterial communities, determining the microbial quorum sensing and intervening in the formation and maturation of oral biofilm. AI-2 of Fn regulates which microbial and/or periodontopathogenic species are inhibited or mature in the biofilm [23]. Most of the microbiome bacteria coexist in a state of symbiosis (eubiosis), but pathogenic bacteria cause states of dysbiosis or pathogenic alteration of the microbiome. Bacterial dysbiosis is linked to different diseases, such as ulcerative colitis, Crohn’s disease, and colorectal cancer [24].

To summarize, Fn is an anaerobic, invasive, and pro-inflammatory bacterium and is one of the most representative pathogenic species of the subgingival microbial environment. It is mainly linked to periodontitis and other infections, and their main pathogenicity mechanisms are the expression of FadA, the expression of Lectin-type Fap-2 molecules, and the AI-2 molecule that mediates its quorum sensing. These capabilities confer the ability to this species to invade host cells, bind to tumoral cells, and shift the local microbiome.

## 3. Colorectal Cancer

**Figure 1:**
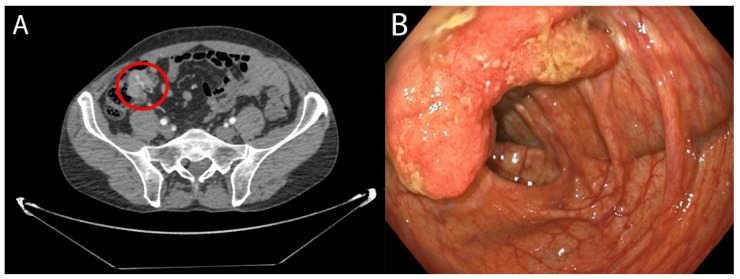
(**A**) Concentric wall thickening of the ileocecal valve compatible with a neoplasia. Approximate measures: 2.8 × 3 × 2.8 cm (T × AP × L). No spiculations on the paracecal fat or regional adenopathy are observed. (**B**) Endoscopic view of tumour. Flat and over-raised lesion with central depression of at least 20 mm in the colonic wall, suggestive of adenocarcinoma.

Colorectal cancer (CRC) comprises a mixed complex of transformed cells with aberrant genomes, non-transformed cells including immune and stromal cells, and often microorganisms (Figure 1, tumour and CT imaging). It is the third leading cause of cancer-related deaths in the United States [25]. Based on cancer records and mortality data from the 27 countries of the European Union, referring to the year 2020, CRC was the second in incidence, with 520,000 cases per year, and the second in mortality, with 250,000 deaths per year [26]. In Spain, CRC was the most diagnosed disease throughout 2020 (44,231 new cases) both in men and women, constituting the second most frequent tumour (26,044 in men and 18,187 in women), behind prostate cancer and breast cancer (35,126 and 32,953 cases, respectively). In addition, in 2018, CRC was the second leading cause of death from tumours (11,265 deaths), only behind lung cancer (22,133 deaths), according to data from the Spanish Society of Medical Oncology of 2020. The main factor in the oncogenesis of this type of cancer is unclear; however, inflammation and the immune response and their products are recognized risk factors. Lately, scientific evidence has increased with respect to the human bowel microbiome as a key factor that influences the development, progression, and response to treatment of CRC [27,28].

The bowel microbiota is the largest reservoir of human microbiota, including at least 1000 different species of known bacteria [29]. Only 150 to 170 species of these bacteria are common to different individuals, most commensals, whose metabolism provides vitamins K and B12, and degrade bilirubin and fibre, providing nutrients that are absorbed [30,31]. A quarter of the weight of faeces is bacteria. Bowel dysbiosis exposes the colon to metabolic and inflammatory stimuli, increases gene mutations, and eventually develops CRC. Species including *Fusobacterium* spp., *Enterococcus faecalis, Bacteroides fragilis, Escherichia coli*, *Parvimonas micra* ATCC 33270, *Streptococcus anginosus*, and proteobacteria were significantly increased in faeces from patients with CRC compared to controls [32].

Incidence and mortality of colon cancer are modifiable thanks to the implementation of effective screening programmes. There are different methods, the most widespread being the detection of occult blood in faeces and colonoscopy, with variable sensitivity and specificity. However, the lack of health resources and the participation and adherence of the population to the different screening programmes are the main drawbacks [33]. There are several risk factors related to the onset of CRC. They can be exogenous, such as smoking, environmental pollutants, obesity, and diet, as well as endogenous, such as genetic mutations and the microbiome [34,35].

Depending on the origin of the mutation, CRC can be classified as sporadic (70%), familial (25%), and hereditary (5%). These mutations cause chromosomal alterations and gene translocations that lead to microsatellite instability, chromosomal instability, and the CpG island methylator phenotype. Mutations affect genes such as c-MYC, KRAS, BRAF, PIK3CA, PTEN, SMAD2, and SMAD4, causing the deregulation of several pathways (MAPK/PI3K, TGF-β, TP53) responsible for cancer progression [35].

CRC can be diagnosed in asymptomatic individuals through population screening programs or in patients who present symptoms or warning signs. The main manifestations that should lead to the suspicion of CRC are constitutional syndrome, gastrointestinal bleeding (rectal bleeding or melena), changes in the depositional rhythm, intestinal obstruction, or chronic iron-deficiency anaemia. The definitive diagnosis is achieved through colonoscopy and biopsy with a histological study [36].

The choice of treatment for CRC follows a multidisciplinary approach, based on the characteristics of the tumours and their staging. The treatment of choice is surgical resection, and neo or adjuvant therapies such as radiotherapy and chemotherapy can be associated. In advanced tumours with distant diseases, the combination of chemotherapy with immunotherapy against the epidermal growth factor receptor (EGFR) or the vascular endothelial growth factor (VEGF) has achieved satisfactory outcomes in terms of increased survival [37].

On the other hand, the modulation of the intestinal microbiota is also a promising strategy to prevent the onset of CRC, to improve the efficacy of treatment, and even to reduce the adverse effects of the therapies [38]. Regarding screening programmes and colonoscopy surveillance, benefits have been documented pointing to reduced risk of colon cancer onset and increased survival rates, given that subsidiary adenomas of definitive endoscopic treatment and early-stage cancers can be detected [39]. Undoubtedly, screening is one of the keys in the prognosis of CRC. It has been observed that patients diagnosed in stage III by the screening programmes had a disease-free survival rate, after five years, of 79.1%, significantly higher than 61.7% of patients diagnosed at this same stage after the appearance of symptoms [40].

**Figure 2:**
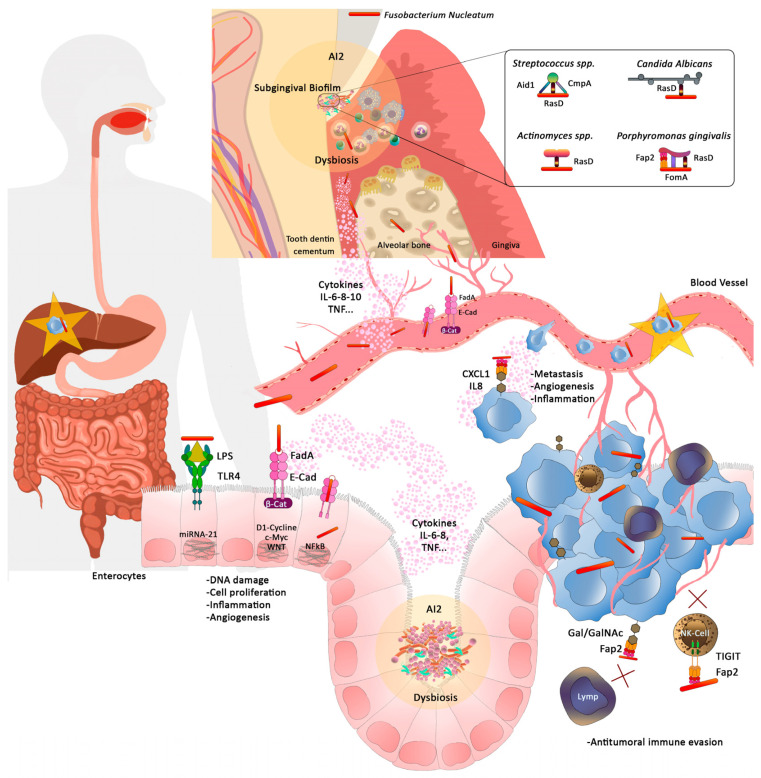
Upper section: *Fusobacterium nucleatum* (Fn) is involved in the formation of the subgingival biofilm by aggregating with primary and secondary colonisers and other species such as *Candida albicans.* through adhesin RasD. It leads the subgingival biofilm towards a dysbiosis state through AI2. The inflammatory process at the gingival level progresses to periodontitis and alveolar bone destruction. Middle section: Fn, through its adhesion molecule FadA, binds to E-cadherin in the endothelial cells of the gingival blood vessels, altering the permeability of these vessels and passing into the bloodstream (indirect diffusion). Pro-inflammatory cytokines from the gingival inflammatory process also pass into the bloodstream. Lower section: Fn in the intestinal lumen through LPS and FadA, activating TLR-4 and β-catenin, induces the synthesis of oncogenes by enterocytes, causing a dysbiosis of the colonic microbiome. In an already developed tumour, it would bind to Gal/GalNAc expressed on the tumour cell surface and evade the anti-tumour immune action of NK-Cell and lymphocytes. Fn can also bind to the metastatic tumour cell and, therefore, travel to other organs such as the liver (star in the graph).

In summary, CRC consists of a group of cancers developed in the colon or rectum and is one of the most important causes of cancer-related deaths. Inflammation and the immune response seem to play a key role in the pathogenesis of these cancers. The colon microbiome is considered as a relevant factor that may influence CRC, and the dysbiosis may increase mutations due to inflammatory and metabolic phenomena of bacterial origin. Therefore, the modulation of colon microbiome may be a relevant approach for the prevention and treatment of these kind of tumours (Figure 2).

## 4. Colorectal Cancer and *Fusobacterium nucleatum*. Pathogenic Mechanisms of the Relationship

It is estimated that between 15 and 20% of cancer worldwide is attributable to known infectious agents. It has been speculated that Fn―given its invasive capacity through digestive transit (direct route) and/or bacteraemia (indirect route)―could function as an oncogenic agent at the level of the colon mucosa. It was also considered that it could aggravate an ongoing tumour process.

In the North American population, Castellarin et al. demonstrated for the first time by quantitative PCR (rtPCR) an unexpected overabundance of Fn in colon tumour samples, 415 times higher than in normal mucosa samples [41]. Kostic et al. performed a genomic analysis of the microbiome in CRC samples and found a significant increase in *Fusobacterium* spp., especially the species *F. nucleatum*, *F. mortiferum*, and *F. Necrophorum*. That analysis also revealed broader changes in the tumour environment, such as the decrease of the phyla *Bacteroidetes* and *Firmicutes*, especially of the order *Clostridiales* [42]. A case-control study that assessed colorectal adenomas showed a statistically significant higher prevalence of *Fusobacterium* spp. in the cases, with a strong positive correlation between higher levels of *Fusobacterium* spp. and the presence of adenomas. In particular, those patients with higher amounts of bacteria had a 3.5-times higher risk of having adenomas. These authors also demonstrated a high correlation between levels of tumour necrosis factor-α and *Fusobacterium* spp. in the mucosa of the colon only in the cases. These authors were the pioneers in demonstrating the physical presence of the bacteria by means of in situ fluorescence using probes of bacterial 16S rRNA [43]. Recent evidence has also confirmed that fusobacteria are able to reach metastases from the primary tumour or from the oral cavity mainly via the circulatory system rather than the gastrointestinal tract [44]. Nishimura et al. demonstrated that Fn might play a role in the proliferation of sessile serrated adenoma/polyp through promoting the β-catenin nuclear translocation and regenerating gene (REG) Iα overexpression [45]. REG is a family of proteins that have a cellular proliferative and antiapoptotic effect on inflamed or neoplastic lesions of the gastrointestinal tract [46].

*Fusobacterium* spp. species may have advantages in the healthy colorectal microenvironment or in evolving tumours, due to low oxygen pressure at the base of the intestinal villi or in the crypts of Lieberkühn. Fn could be incorporated into the intestinal biofilm and favour a dysbiotic microbiota according to the alpha-bug hypothesis. According to Sears and Pardoll, the concept of the alpha-bug (alpha species) refers to selected members of a microbial community. In addition to exhibiting virulence and procarcinogenic characteristics, they are capable of remodelling the microbiome to drive a pro-inflammatory immune response and favour cellular transformation of the colon epithelium that leads to cancer [47]. Other authors call this same hypothesis ‘keystone pathogens’ [48]; however, although it makes sense, this hypothesis is not conclusive, since bacteria that are considered pathogens may be present in a healthy individual and not behave as pathogens [49].

Evidence has been provided with respect to the possible mechanisms occurring in the oral microbiome in CRC. Flanagan et al. performed qPCR in CRC biopsies and observed a higher amount of Fn in comparison to adenomas and healthy tissue samples from the European population. However, adenomas with a higher degree of dysplasia were significantly associated with higher amounts of Fn. These authors also showed that CRC patients with lower Fn levels had higher survival rates in comparison to patients with higher levels of the bacteria. Another goal of these authors was to determine whether Fn would behave as a possible non-invasive biomarker for CRC screening, showing that, although Fn was more abundant in faeces samples from patients with CRC compared to adenomas or controls, the bacteria in faeces did not correlate with the grades of cancer or adenomatous tissue [50].

Rubinstein et al. showed that Fn invaded both normal and tumour epithelial cells via FadA─E-cadherin binding, stimulating the growth of tumour cells but not healthy epithelial cells. In tumour cells, FadA─E-cadherin activated the b-catenin transcriptional pathway, resulting in increased expression of cyclin D1, c-myc, and Wnt oncogenes. Furthermore, through its lipopolysaccharide and the nuclear factor kappa-β transcriptional pathway, Fn encoded the synthesis of pro-inflammatory genes IL-6 and IL8, both pro-inflammatory cytokines, and carcinogenic markers [51]. The same authors have shown in recent results for the time the capacity of Fn to selectively stimulate the growth of colorectal cancerous cells through the role of Annexin A1 [52].

FadA is unique to Fn and could have a potential use as an early marker of CRC risk. Its levels can also be related in a diagnostic way to health conditions, pre-cancer, or cancer. Through Fap2 lectin, Fn has the ability to recognize and bind Gal-GalNAc, expressed by CRC cells and other tumours. High levels of Gal-GalNAc have been detected in CRC metastases and were correlated with high Fn levels [22]. Once in the tumour, Fn could accelerate cancer development by promoting cell proliferation, creating a favourable inflammatory environment and/or blocking the action of NK cells or T lymphocytes against the tumour. In the sense of progression, recently, X Li et al. demonstrated how, through cyclin-dependent kinase 5, Fn activated Wnt/β-catenin signalling to modulate CRC progression [53]. In the sense of the relationship with specific immunity, recently, Boroswky et al. demonstrated how Fn was associated with lower stromal density in tumour tissue of CD3+ lymphocytes (T helper lymphocytes) [54].

The distinction between molecular subtypes of CRC has also produced interesting results. According to the results obtained by Purcell et al., the microbiological analysis of the different molecular subtypes of CRC showed different combinations of bacterial species and some of oral/periodontal origin in addition to Fn, such as *Porphyromonas gingivalis* and *Prevotella* spp. This finding reaffirms the theory of the role played by the oral microbiome in the development of CRC [55], together with the fact that 40% of the patients showed identical Fn strains in CRC samples and in saliva samples [56]. On the other hand, the consumption of a diet rich in whole grains and dietary fibre has recently been associated with a lower risk of suffering from CRC colonized by Fn. Therefore, factors such as diet can also modify intestinal colonization by Fn [57].

Yang et al. found that the infection of CRC cell lines with Fn increased proliferation, invasive activity, and the ability to form new tumours in mice. Fn activated TLR-4 and increased the expression of MicroRNA-21 (mR-21), showing a possible epigenetic effect on tumour cells. Mice with higher levels of Fn and mR-21 had shorter survival rates [58]. This was the first experimental study that has demonstrated the oncogenic capacity of Fn. Casasanta et al. showed that Fn may both directly and indirectly modulate the immune modulation and signalling of CRC by inducing the secretion of pro-inflammatory cytokines, such as IL-8 and CXCL1, associated with migration, poor diagnosis, and metastatic potential [59].

MicroRNAs are non-coding molecules of RNA that regulate target gene expression negatively. It has been shown that oncogenic miRNAs, such as miR-21, miR-224, miR-200c, miR-96, miR-135, miR-31, and miR-155, were related to the pathogenesis of CRC [60,61]. Fn showed the capacity to target innate immunity receptors and specific micro-RNAs, activating the autophagy pathway, and therefore inducing CRC chemoresistance [62].

A recent finding correlated Fn with high glucose metabolism in CRC patients, which occurred by activating the transcription of long non-coding RNA enolase1-intronic transcript 1 (ENO1-IT1) [63]. ENO1 is a protein expressed in the cell membrane that performs multiple functions such as catalysing intermediate steps in glycolysis and plasminogen synthesis, stimulating cell migration and invasion, or binding to DNA, mRNA, and lncRNA to regulate the transcription and translation of cancer cells [64]. ENO1 is significantly overexpressed in CRC tissues.

Bullman et al. found that Fn and its associated microbiome, including Bacteroides, *Selenomonas,* and *Prevotella* spp., were present not only in primary CRC, but also at a distance, within the metastatic cells in liver rather than in the stroma. Treatment with metronidazole to reduce Fn load in mice infiltrated with Fn-positive CRC cells stopped tumour growth by 30%. The authors concluded that this outcome was consistent with a causal role of the bacterium in tumorigenesis [65]. That study published by Bullman et al. in Science of December 2017 and that of Yang et al. in Gastroenterology, also from 2017, were the first to demonstrate the oncogenic capacity of Fn. Both were based on results obtained in animal models and on the basis of the existence of tumour cells infected with Fn. However, this relationship has not only been described in situ, but also in faeces samples in which the abundance of Fn was observed, with an increased risk of being diagnosed with rectal cancer of up to five times higher [66].

The first meta-analysis addressing this relationship was published in 2018. It was conducted with seven studies published until 2017 and involved 1198 participants (629 CRC patients and 569 healthy controls). It assessed whether intestinal infection by Fn (isolation of the bacteria) could be considered a diagnostic marker for CRC. The ROC curves of the study showed a sensitivity of 81% (95% CI = 0.64–0.91), specificity of 77% (95% CI = 0.59–0.89), and odds ratio of 14.00 (95% CI = 9.00–22.00). The authors concluded that Fn was a valid marker for the diagnosis of CRC, but it remained to be proven whether this method would be more efficient than known diagnostic strategies [67]. Until October 2020, Janati et al. performed a meta-analysis with twelve studies to determine the epidemiological evidence of the association between Fn and CRC. The results showed a positive association between Fn detection in colorectal specimens and CRC (OR = 8.3; 95% CI = 5.2–13.0) [68]. On the other hand, Huangfu et al., also up to April 2020, performed a meta-analysis of 13 selected studies, including 3626 patients with CRC. These authors concluded that high levels of Fn were associated with poor prognoses, tumour growth, distant metastasis, poor differentiation, tumour-associated genes (KRAS mutation), and high microsatellite instability in CRC patients [69]. The last meta-analysis on this topic has been recently published by Xuan et al., with 14 studies involving 634,744 participants, reporting that periodontal patients were 21% (95% CI 1.06–1.38) more likely to develop CRC compared to periodontally healthy patients, but their results showed no significant association between periodontitis and CRC mortality [70].

Briefly, infectious agents may be agents for the development of cancers. Among them, Fn has been extensively related to CRC in the literature. Fn has shown to be able to access and grow in the tumor site directly though the digestive tube or indirectly disseminating through the bloodstream. The main consequences of this infection are the ability of Fn to invade colon epithelial cells and activate oncogenesis or contribute to tumor progression. These bacterial infections have been also reported in the metastases of CRC, reinforcing the dissemination theory of these bacterial species. 

## 5. Conclusions and Future Perspectives

There is very recent and strong evidence relating Fn to CRC. However, there are still unknown aspects that need to be elucidated. We need to know whether Fn colonizes directly (direct route) or through blood (indirect route); how it reaches the intestinal lumen; the oncogenesis phase in which it would be involved, i.e., onset, promotion, development, or extension of the tumour; and whether the colonization is the result of the tumour or intestinal inflammation. At the intestinal level, the bacteria would find an ideal microenvironment to cause disruption of the balance between the local microbiota and the immune system, with dysbiosis as a consequence. An intestinal dysbiotic biofilm can cause intestinal diseases such as inflammatory bowel disease, irritable bowel syndrome, and CRC.

Possibly, the most powerful theory is the one considering the development of an inflammatory process with chronic exposure to inflammatory mediators, activation of oncogenes, and development of CRC. Fn is a native species of the oral cavity, more specifically, very abundant in the subgingival biofilm of patients with periodontitis, given the connectivity of the digestive tract. If it is through that tract that intestinal colonization occurs, then it will be necessary to determine how the bacterium is capable of crossing an adverse gastric environment that is acidic (pH = 0 or 1) and in which only another gram-negative bacterium (Helicobacter pylori) can survive thanks to its positive urease activity. Indeed, Helicobacter pylori is, to date, the only recognized oncogenic bacterium (55).

In addition, tumours induce angiogenesis, increased vascular permeability, hypoxia, and local immunosuppression, which are non-specific local factors that would help oral bacteria colonize CRC through blood. Issues such as the following are some of the uncertainties that further prospective research in humans should clarify:

(a) Is periodontitis a risk factor for CRC or other inflammatory bowel diseases?
(b) Is it through the bloodstream or the digestive tract that Fn interacts with the enterocyte?
(c) Through FadA (given its antigenicity), the other fusobacterial adhesins that it exhibits, and the intracellular transcriptional pathways that it activates, is Fn another oncogenic bacterium? If it is, in what tumour phase would it have an effect?
(d) Would oral (periodontal treatment) and systemic (probiotics, prebiotics, and/or antibiotics) Fn-reducing treatment or manipulation of intestinal microbiota through faecal implants or vaccines have an impact on relapse-free survival and overall survival?
(e) Would new prospective studies confirm the role of Fn in promoting metastasis and antitumour immunity evasion in CRC?
(f) Since Fn and glucose metabolism are related to CRC, can targeting the ENO1 pathway be a strategy in the treatment of patients with CRC and elevated Fn?
(g) Could Fn oncomicrobiotics (cocktail of Fn or its products) be used as a novel approach to improve the immune response to CRC?

## Data Availability

Not applicable.
